# Prediction of Myocardial Infarction From Patient Features With Machine Learning

**DOI:** 10.3389/fcvm.2022.754609

**Published:** 2022-03-14

**Authors:** Zhihao Chen, Jixi Shi, Thibaut Pommier, Yves Cottin, Michel Salomon, Thomas Decourselle, Alain Lalande, Raphaël Couturier

**Affiliations:** ^1^FEMTO-ST Institute, UMR 6174 CNRS, Univ. Bourgogne Franche-Comté, Belfort, France; ^2^IRSEEM, EA 4353, ESIGELEC, Univ. Normandie, Saint-Étienne-du-Rouvray, France; ^3^Department of Cardiology, University Hospital of Dijon, Dijon, France; ^4^CASIS Company, Quetigny, France; ^5^Department of Medical Imaging, University Hospital of Dijon, Dijon, France; ^6^ImViA Laboratory, EA 7535, Univ. Bourgogne Franche-Comté, Dijon, France

**Keywords:** acute myocardial infarction, automatic prediction, clinical characteristics, DE-MRI, machine learning

## Abstract

This study proposes machine learning-based models to automatically evaluate the severity of myocardial infarction (MI) from physiological, clinical, and paraclinical features. Two types of machine learning models are investigated for the MI assessment: the classification models classify the presence of the infarct and the persistent microvascular obstruction (PMO), and the regression models quantify the Percentage of Infarcted Myocardium (PIM) of patients suspected of having an acute MI during their reception in the emergency department. The ground truth labels for these supervised models are derived from the corresponding Delayed Enhancement MRI (DE-MRI) exams and manual annotations of the myocardium and scar tissues. Experiments were conducted on 150 cases and evaluated with cross-validation. Results showed that for the MI (PMO inclusive) and the PMO (infarct exclusive), the best models obtained respectively a mean error of 0.056 and 0.012 for the quantification, and 88.67 and 77.33% for the classification accuracy of the state of the myocardium. The study of the features' importance also revealed that the troponin value had the strongest correlation to the severity of the MI among the 12 selected features. For the proposal's translational perspective, in cardiac emergencies, qualitative and quantitative analysis can be obtained prior to the achievement of MRI by relying only on conventional tests and patient features, thus, providing an objective reference for further treatment by physicians.

## 1. Introduction

Acute myocardial infarction (MI) has become one of the most common cardiovascular diseases in the emergency department (1). MI occurs as a result of myocardial distress leading to the death of myocardial tissue. In the clinical context, MI is usually due to thrombotic occlusion of a coronary artery, most commonly caused by the rupture of an atherosclerotic plaque. Ischaemia induces perturbations in the myocardium and leads to a rapid depression of cardiac functions, and then in cases of prolonged ischaemia, necrosis of the myocardial tissue may occur. If the revascularization is delayed or fails, the extensive damage can lead to persistent microvascular obstruction (PMO), also known as the no-reflow phenomenon (2). Therefore, emergency revascularization therapy to restore perfusion is crucial as soon as the disease is diagnosed.

**Figure 1:**
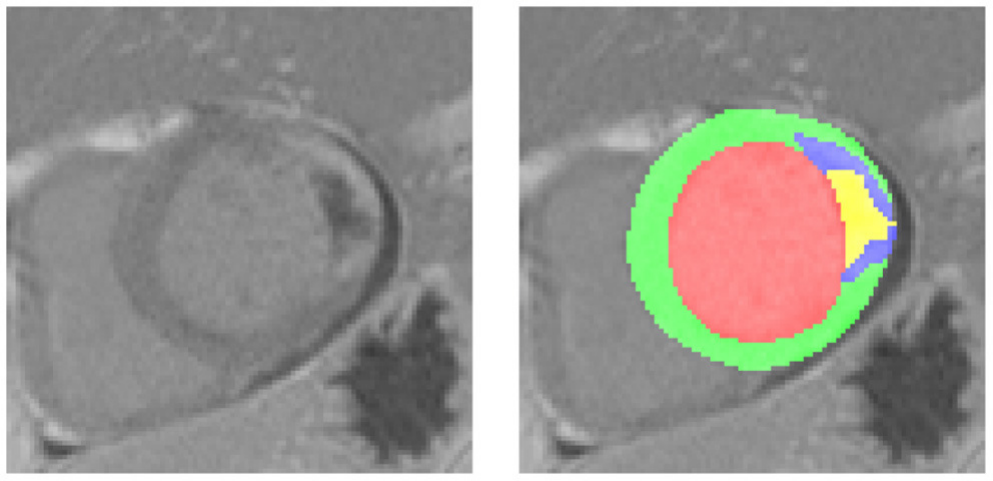
A typical Delayed Enhancement MRI (DE-MRI) slice involving the myocardial infarction (MI) and the persistent microvascular obstruction (PMO). On the left the cropped short-axis DE-MRI covering the whole left ventricle, on the right the corresponding masks of normal myocardium (green), MI (blue, PMO exclusive) and PMO (yellow). The PMO is semi-wrapped by the MI and contacts the cavity.

A variety of medical diagnosis methods can be proposed to detect or evaluate the extent of acute MI. However, the accuracy is often in conflict with the time required for the diagnosis, which remains a therapeutic emergency. For example, the cardiac Delayed Enhancement MRI (DE-MRI) is the gold standard for the diagnosis and evaluation of MI (3). Indeed, DE-MRI can precisely indicate the severity of MI, especially in the area of cardiac necrosis. The infarct area has usually higher intensity than the normal myocardium due to the difficulty in draining the contrast agent in time. The PMO can be characterized by the low intensity area wrapped by the infarct and touching the endocardium (4). Figure 1 shows one DE-MRI slice involving both the infarct and the PMO and their annotations. Nevertheless, the MI diagnosis with DE-MRI cannot be widely applied in the emergency department because of its required time for the acquisition and post-processing. In current practice, simple tools such as ECG, troponin assay, and echocardiography are used to validate the emergency diagnosis of MI. ST segment analysis on ECG (especially in case of ST persistent elevation), the intensity of troponin elevation, or LVEF (left ventricular ejection fraction) assessment from transthoracic echocardiography (TTE) have been shown highly correlated with MI (5–8). Given these facts, when patients arrive in the emergency department complaining of chest pain, generally a series of indicators will be first listed with the help of the above-mentioned simple tools. If the examinations reveal the possibility of MI, the DE-MRI could be achieved the next days to have a more accurate evaluation of the myocardial impairment, after the acute phase and the early therapeutic management including revascularization and medications. Until the ultimate diagnosis based on the MRI exam is available, physicians mainly rely on the obtained physiological, clinical, and paraclinical characteristics to determine the severity of a patient's condition and to give sound treatment advice.

In order to better interpret these data, studies on the correlation between the physiological, clinical, and paraclinical data, and the symptoms of MI have been conducted for decades. More than 30 years ago, Goldman et al. (9) developed a computer protocol to diagnose the related diseases when a patient received by an emergency department complained of chest pain unexplained by trauma or chest-film abnormalities. Their decision protocol was based on a recursive partitioning approach (10). About 50 potential predictive variables from the clinical history of the patient, physical examination, and ECG were incorporated in to the decision protocol and the ultimate diagnosis of MI depended on three criteria namely the serum enzyme level, the comparison of the Q and R waves with the first ECG, and the cardiac scintigraphy. In the case of sudden unexplained death within 72 h of receiving the patient, Than et al. (11) also predicted the likelihood of acute MI with decision trees afterward. Their prediction model incorporated the age, sex, and serial cardiac troponin 1 concentrations, and the ultimate diagnosis was adjudicated according to the universal definition of MI (12). Similarly, Romero-Farina et al. (13) tried to predict the risk score for cardiac events. The gated SPECT metrics were considered with other clinical features for the prediction, which is the major highlight of the study.

Other studies were dedicated to developing classification models of the presence of MI or other cardiac events. Al-Zaiti et al. (14) made the acute coronary syndrome prediction with temporal-spatial features of the 12-lead ECG. This study aimed at increasing the quality of pre-hospital ECG diagnosis. Faced with a huge number of features, Gárate-Escamila et al. (15) applied Principal Component Analysis (PCA) to reduce the dimensionality of the data, Daraei et al. (16) used Evolutionary Algorithms for the feature selection, then they classified the heart disease with Machine Learning models. Unlike the features studied in previous articles, Melillo et al. (17) proved that the heart rate variability-based classifier showed higher predictive values than the conventional echographic parameters for the cardiovascular event prediction.

To summarize the above-related studies, most of them aimed at predicting the likelihood or classifying the presence of MI and related cardiac events. Especially in pre-hospital or emergency cases, automatic predictive models have been shown to be of great clinical relevance. However, few researchers have attempted to develop predictive models of MI based on clinical features in conjunction with medical imaging. Moreover, few studies mentioned the automatic assessment of the PMO given that its clinical diagnosis mostly relies on invasive or imaging techniques (4). The review of the literature reveals that no study has tried to train a predictive model through the quantitative data of MI provided by Cardiovascular MRI (CMRI) to obtain a prediction of its severity.

In the light of the above-mentioned facts, an automatic prediction approach is proposed to precisely classify and quantify the severity of the acute MI only taking into account the physiological, clinical, and paraclinical features. Furthermore, experimental attempts have also been made to estimate the PMO individually with the same approach. The predictions are based on standard machine learning algorithms involving linear model, Random Forest (RF) and Decision Trees, Support-Vector Machines (SVMs), Multilayer Perceptron, and boosting models. For each patient, the annotated DE-MRI provides the quantitative ground truth of the infarction and the selected patient features are thought as the input data. For the training stage, the features are the inputs of an appropriate machine learning model. Depending on the classification or the quantification task, the state of the myocardium or the Percentage of Infarcted Myocardium (PIM) calculated from the DE-MRI are the target output of the model. For the inference stage, once the model is well-trained with the paired patient features and DE-MRI, it can predict the severity of the patient's infarction only according to the patient features. The investigated data for the experiments come from the automatic Evaluation of Myocardial Infarction from Delayed-Enhancement Cardiac MRI (EMIDEC) Challenge (that took place in 2020) database, which consists of 150 cases of paired physiological, clinical, and paraclinical features, and annotated DE-MRI (18). It is important to note that the proposal aims at providing early prediction to better guide patients in the emergency department. Therefore, the automatic prediction should only be used as an aid to clinical diagnosis and the associated risk of mispredictions should be taken into account by physicians.

## 2. Methods

### 2.1. Ethics Approval

The dataset was collected from daily clinical exams at the University Hospital of Dijon, France. All the data were fully anonymized and handled within the regulations set by the local ethical committee. The ethical committee of the University Hospital of Dijon checked the compliance of the dataset in accordance with the Declaration of Helsinki.

Because of the NIfTI (Neuroimaging Informatics Technology Initiative) data format for the MRI and the ambiguity of the patient features, the retrospectively collected data were completely untraceable. According to the French law and the ethical committee of the University Hospital of Dijon, neither the ethics committee approval nor the informed written consent was required.

### 2.2. Patient Features

The data, consisting of 150 exams, were collected at the University Hospital of Dijon, France in the framework of the EMIDEC Challenge (18). All the patients included in the dataset presented symptoms suggestive of MI at the emergency department. The number of infarcted and non-infarcted cases was not balanced, which reproduced the clinical background of the hospital. Physiological, clinical, and paraclinical data were examined during the arrival of the patients. Twelve indicators that are potentially related to acute MI were selected to compose the patient features. Table 1 shows the characteristics of the selected features and Table 2 lists the statistics of MI and PMO among pathological subjects.

**Table 1:** Characteristics of pathological and non-pathological patients [according to the Delayed Enhancement MRI (DE-MRI)].

**Patient feature**	**Non-pathological subjects (*n* = 50)**	**Pathological subjects (*n* = 100)**	***p*-value^**d**^**
Sex	38 females and 12 males	23 females and 77 males	0.000
Age	66 ± 14 years	59 ± 12 years	0.004
Tobacco (yes, no, former smoker)	18%, 22%, 60%	44%, 21%, 35%	0.001
Overweight^a^	62%	53%	0.296
Arterial hypertension	58%	31%	0.002
Diabetes	20%	10%	0.126
History of coronary artery disease	4%	12%	0.065
ECG (ST elevation)	30%	80%	0.000
Troponin (ng per mL)	7.68 ± 12.91	101.04 ± 101.35	0.000
Killip max (1,2,3,4)	76%, 22%, 2%, 0%	83%, 12%, 2%, 3%	0.916
LVEF^b^ (percentage)	49.62%±13.49%	47.74%±13.17%	0.423
NTProBNP^c^ (pg per mL)	2, 136 ± 3, 696	1, 314 ± 2, 109	0.154

*^a^If BMI >25.*

*^b^Left Ventricular Ejection Fraction, calculated from transthoracic echocardiography.*

*^c^N-terminal pro-B-type natriuretic peptide.*

*^d^p-value of independent t-test*.

**Table 2:** The proportion of scar tissues among pathological subjects.

**Pathological tissue**	**PIM**	**Presence (%)**
MI (PMO inclusive)	0.1825 ± 0.1152	100
PMO	0.0330 ± 0.0360	51

Cardiovascular MRI was then performed several days after the arrival of the patient, containing DE-MRI in short-axis orientation covering the left ventricle thanks to a T1-weighted phase-sensitive inversion recovery (PSIR) sequence. The exams were done on 1.5T and 3T magnets (Siemens Medical Solution, Erlangen, Germany) with a phased thoracic coil. After the image acquisition, the contours of the left ventricular myocardium as well as the infarction and PMO areas were drawn by experienced doctors with the help of the commercial software QIR. PIM was, thus, calculated from the manual annotations to describe objectively the severity of the MI.

It should be noted that in this study, the term pathology corresponds to MI. The pathological subjects include patients with MI confirmed *via* DE-MRI assessment, and the non-pathological subjects may suffer from cardiac diseases other than MI (with normal DE-MRI).

### 2.3. Study Design

**Figure 2:**
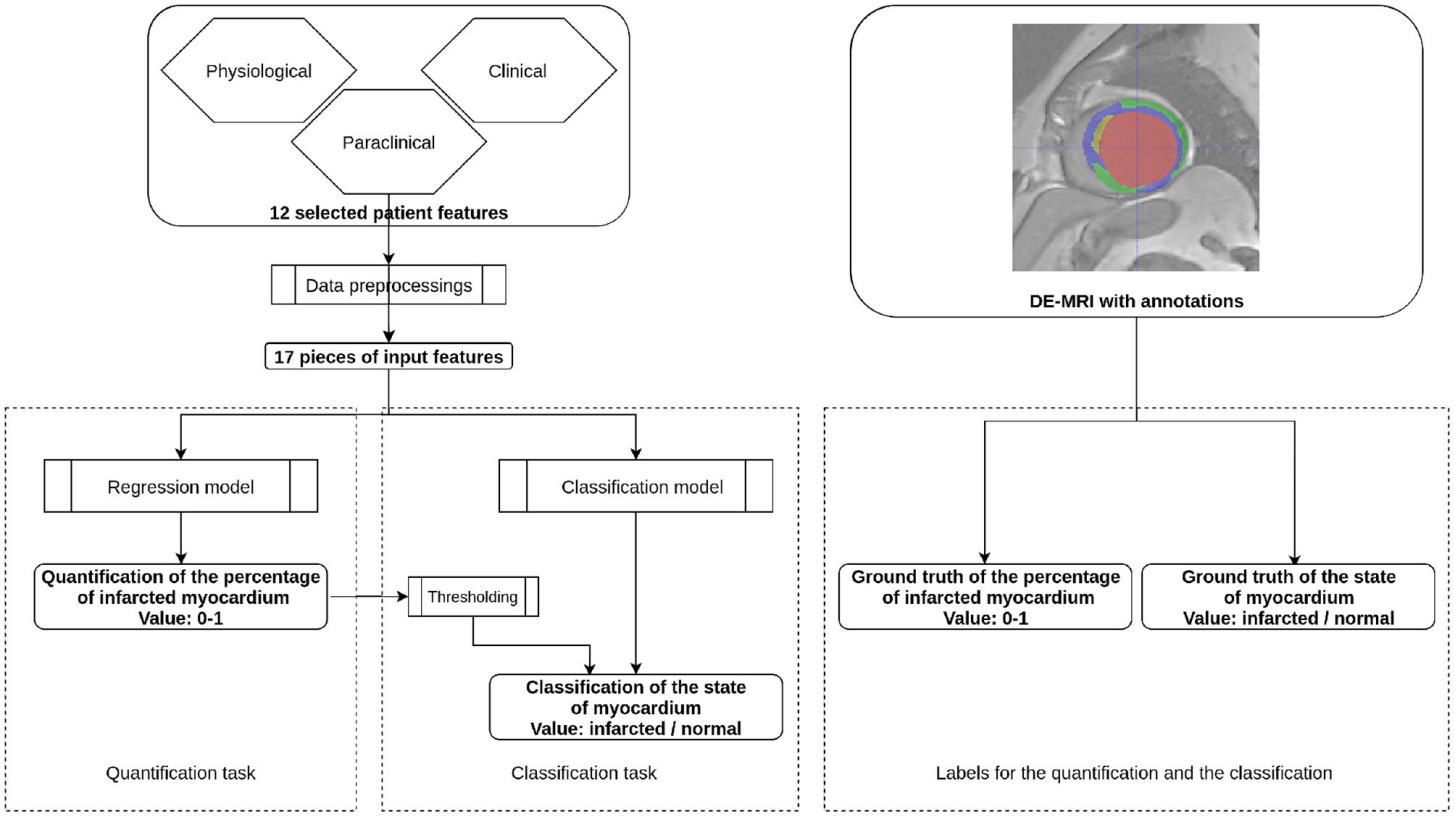
Workflow of the proposal. This figure presents the inference stage for the quantification task and the classification task of the automatic MI prediction. On the left part, selected patient features are first preprocessed to 17 pieces of numerical or Boolean features. For the quantification task, the features are incorporated through a regression model so that the obtained value is the Percentage of Infarcted Myocardium (PIM) ranging from 0 to 1. In the case of the classification task, the prediction can be obtained by either a regression model followed by thresholding or a classification model. During the training stage, the regression model is supervised by the ground truth PIM, and the classification model is supervised by the ground truth state of the myocardium. Both ground truths are defined from the DE-MRI and manual annotations.

Two prediction problems were targeted: the quantification of PIM and the classification of the state of the myocardium. Predictions were carried out with classification and regression algorithms. Each training case incorporated the 12 patient features shown in Table 1, and the PIM or the state of the myocardium was evaluated from the DE-MRI and its annotations. The machine learning models were first trained with paired input data, i.e., patient features and the ground truth. During the inference stage, only the clinical features were fed to a trained predictive, model and the model's output was the predicted PIM or the state of the myocardium. Figure 2 shows the workflow of the proposal during the inference stage.

#### 2.3.1. Data Preprocessings

Physiological, clinical and paraclinical data should be first preprocessed so that the machine learning models can manage the features correctly. The data format of the model's input should be numerical, therefore, the categorical features, i.e., Tobacco and Killip max were converted to one-hot encoding and Boolean features were encoded as 0 or 1. Gradient descent-based and distance-based algorithms are sensitive to feature scaling. Therefore, normalizing the features to a universal interval may improve the predictive performance of such models (19). To address this fact, in addition to the numerical encoding, normalization was also applied on the features of age, troponin, LVEF, and NTProBNP.

Being the ground truth of the machine learning models for the quantification task, the PIM was calculated from the manual annotations of myocardium and scar contours. Given that the voxel size is constant for each DE-MRI case, the calculation of the volumes of myocardium and infarcted areas only relied on the manually drawn contours of all the myocardial tissues. Thus, the PIM of one patient was calculated as


(1) $$\begin{array}{l} \text{PIM}=\text{Volume}\left(\text{scartissue}\right)/\text{Volume}\left(\mathrm{myocardium}\right) \end{array}$$

where the volume referred to the voxel quantity of scar tissue in the DE-MRI case. The scar tissue could be either MI or PMO. For the MI evaluations, the PMO was considered as a part of the MI area. The PIM value, which was theoretically ranged from 0 to 1, described the severity of MI. For the classification task, the state of the myocardium denoted the presence of the infarction. Therefore, the data format of the state of the myocardium was Boolean: the MI or the PMO was detected or not from the DE-MRI.

#### 2.3.2. Machine Learning Algorithms

The employed predictive models included regression models and classification models. Regression models estimated the relationships between the dependent variable of numerical values, i.e., the predicted PIM and the input patient features, while for the classification models, their dependent variable was the binary state of the myocardium. For the quantification task, regression models should be applied since the expected output was the PIM. Therefore, the target label during the training stage for the regression model was the calculated PIM according to the DE-MRI. For the classification task, both regression and classification models were feasible. Indeed, a regression model followed by thresholding could also provide a binary classification result of the state of the myocardium.

For the regression task, a linear model was first studied. Linear regression tries to establish a linear function that links the input features and the regression label (20, 21). Although the linear model can predict the PIM with the scalar patient features, non-linear models may be of more interest since the PIM regression task may be a non-linearly separable problem due to the complexity of the input features. Therefore, more learning algorithms using non-linear models including SVMs with non-linear kernel function, Random Forest, and Decision Trees, Multilayer Perceptron, and boosting models were investigated. SVM is not necessarily a non-linear model, however, with a non-linear kernel function that maps the data to a higher dimension, the SVM can solve the non-linearly separable problem (22). The decision Trees algorithm has a flowchart-like structure that consists of nodes (23). Optimized from Decision Trees, RF is trained on uncorrelated Decision Trees as its name suggest, and the inference is made by the individual trees. RF is by definition more robust to overfitting so it generally outperforms Decision Trees (24). Boosting methods are the ensemble of sequentially connected weak learners (25). For example, Gradient Boosting Decision Trees consist of a series of trees, which are the weak learners in this boosting method. Errors are passed between trees, with each tree attempting to reduce the errors passed from the previous tree (26). Gradient Boosting Decision Trees algorithm tend to outperform RF in practice, however, the sequential structure results in its longer computation time than the parallel structure in RF. Multilayer Perceptron is a kind of feedforward artificial neural network. Inputs are passed through multiple layers in which data are mapped with non-linear activation functions in the forward stage (27). Moreover, knowing that a regression model and a classification model could share the same learning algorithm but different optimization functions, the widely used SVM (with linear kernel function) and RF were selected as the learning algorithm for the classification models.

In addition to the predictions obtained from an individual model, the ensemble method was also investigated. The ensemble method is used to improve the predictive reliability by combining the predictions from all individual models into a single set of predictions (28). In this study, for each prediction target, the ensemble method's prediction was derived by adding up then averaging all the models' predictions.

### 2.4. Statistical Analysis

#### 2.4.1. Primary Analysis

The quantitative prediction of the severity of the MI, i.e., the quantification of the PIM including MI (PMO inclusive) and PMO (MI exclusive), is the main objective of the study. The absolute scar tissue volume, which is another possible severity indicator, is not adopted since its severity evaluation can be biased by the patient's physiological conditions. The proposal's performance can be described as the absolute quantification error between the inference result of machine learning models and the PIM calculated from DE-MRI for each scar tissue. Multiple machine learning-based regression models were compared with the ground truth PIM. The comparison results are presented as the mean absolute difference. In order to make the best use of the available data, given the limitations of available data cross-validation was used for the experiment evaluations.

#### 2.4.2. Secondary Analysis

In addition to the quantitative analysis, a qualitative analysis was also performed as the secondary analysis, i.e., the classification of the state of the myocardium. As in the primary analysis, MI and PMO are the two assessed scar tissues. The classification could be carried out by both classification and regression models. However, the data formats of the training labels and the predicted values were different when both the models were applied for the classification task. The classification carried with regression models consisted of the regression models and thresholding. The regression models were the same as in the primary analysis: the training was supervised with the PIM, therefore, the model's output was the PIM. A discrimination threshold differentiated if the patient was pathological from the prediction of the regression model. Differently, when the classification was carried out with classification models, the classification models were trained with Boolean target labels which annotated if the case was normal or pathological. Therefore, the predictive value of the classification models was also Boolean. Both the classification methods can be referred in Figure 2.

For classification models, the classification performance could adequately be evaluated by the sensitivity (or recall), specificity (or selectivity), precision, and accuracy metrics. However, the discrimination threshold used in the regression model could impact the confusion matrix, thus, the Receiver Operating Characteristic curve (ROC) was also adopted to plot the true positive rate (TPR) against the false positive rate (FPR) to intuitively compare between classification and regression models by considering the Area Under Curve (AUC) (29).

#### 2.4.3. Additional Analyses

Additional analyses were investigated under the contests of the quantification of the PIM and the classification of the state of the myocardium. Training database size is crucial for machine learning models. Therefore, the quantification performance with different training data volumes was compared. Then the quantification error was studied according to the severity of MI with the help of Bland–Altman plot (30). Moreover, the importance of individual features for different predictive models was extracted. The quantification of scar tissues with limited selected features was also investigated. Furthermore, our results were compared with the type of MI defined from the fourth universal definition of MI (only type 1 and type 2 were considered) (31). First, the correlation between the classification results and the type of MI was done in detail. Then, the mean PIM was calculated per type of MI. Finally, cases that had important inconsistencies between the prediction and the ground truth were listed to undertake further medical interpretations.

## 3. Results

The machine learning models were implemented with scikit-learn (32), XGBoost (33) and lightGBM (34) libraries, and Python 3.6.9. The training and the inference were only conducted with CPU and since the operation time was in the order of seconds, the computational time was not specifically listed. The experiments were conducted on the EMIDEC Challenge (2020) dataset and the trained regression and classification models are accessible from Github^1^. Except for the tests of the training set volume in Section 3.3, all other experiments employed 10-fold cross-validation, i.e., the training set volume of each split was 135 patients.

### 3.1. Regression Models for the PIM Quantification

The performance of different regression models are presented in Table 3. Linear regression model of the ordinary least squares and other non-linear regression models were evaluated. The ensemble of all the models' prediction was also examined. Using the mean PIM calculated from the ground truth as the predicted PIM, the assumed quantification was achieved as the baseline.

**Table 3:** Prediction error of regression models for the PIM quantification.

**Regression model**	**Predicted PIM error**	
	**MI^**a**^**	**PMO**
Linear Regression	0.0639 ± 0.0677	0.0152 ± 0.0214
Support Vector Regression	0.0579 ± 0.0632	**0.0116** **±0.0238**
Decision Tree Regressor	0.0679 ± 0.0741	0.0162 ± 0.0293
Random Forest	0.0587 ± 0.0597	0.0149 ± 0.0227
Multilayer Perceptron	**0.0578** **±0.0609**	0.0179 ± 0.0229
Gradient Boosting Regressor	0.0602 ± 0.0584	0.0152 ± 0.0228
XGBoost	0.0646 ± 0.0572	0.0172 ± 0.0199
Light Gradient Boosting	0.0590 ± 0.0616	0.0161 ± 0.0227
Ensemble	0.0555 ± 0.0594	0.0141 ± 0.0210
Mean predicted PIM^b^	0.1070 ± 0.0693	0.0162 ± 0.0206

*In bold is the best result of a single model. The ensemble is the average predictions of all the regression models.*

*^a^PMO inclusive. ^b^Calculated from the ground truth*.

Multilayer Perceptron and SVM (with non-linear kernel function) respectively obtained the lowest mean PIM difference for the MI and PMO prediction, and RF achieved relatively low mean quantification error with a small variance. Results also revealed that the ensemble of all the prediction outperformed each single regression model for the MI quantification. The satisfying regression performance of SVM and Random Forest and their much shorter processing time compared to Multilayer Perceptron justified the choice of experimental learning algorithms for the classification task. RF would be performed in all following experiments concerning the classification, SVM would be employed only for the classification in the analysis of the importance of patient features.

### 3.2. Classification of the State of the Myocardium

**Figure 3:**
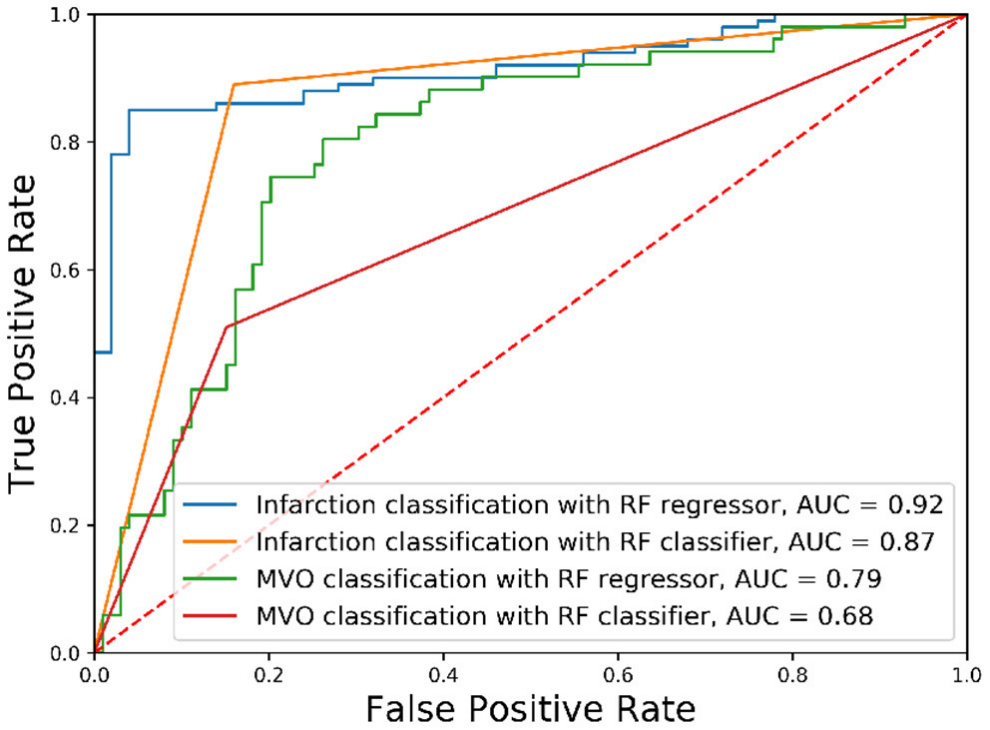
Receiver operating characteristic curves of classification results. The classification of different tissues was realized with Random Forest (RF) algorithms with different optimization functions.

The classification of the state of the myocardium was performed on both the presence of MI and PMO in two ways. Figure 3 shows the classification results of the presence of the infarction and the PMO. The results shown in Table 4 presents the statistical metrics of the same methods as in Figure 3. For the thresholding, the best threshold value was obtained by iterating from 0 to 1 with a step of 0.001 and observing the best accuracy. Table 4 revealed that with the regression model and thresholding, the infarction classification error mostly resulted from the false-negative predictions according to the relatively low recall. Moreover, classification on the ground truth of MI and PMO with the retained threshold values, the sensitivity of the infarction classification was correct (87.00%) while many cases suffering from PMO (sensitivity = 56.86%) may be omitted. It also implied that with the classification report, physicians should pay particular attention to the missed suspected patients in case of negative prediction.

**Table 4:** Classification results under different metrics.

**Prediction model**	**Target tissue**	**Sensitivity**	**Specificity**	**Precision**	**Accuracy**
RF Regressor^a^	Infarction, θ = 0.064	85.00%	96.00%	97.70%	88.67%
	PMO, θ = 0.013	70.59%	80.81%	65.45%	77.33%
RF Classifier	Infarction	89.00%	84.00%	91.75%	87.33%
	PMO	50.98%	84.85%	63.41%	73.33%
RF Classifier with only ECG and troponin	Infarction	77.00%	62.00%	80.21%	72.00%
	PMO	58.82%	79.80%	60.00%	72.67%
GT with thresholding	Infarction, θ = 0.064	87.00%	100.00%	100.00%	91.33%
	PMO, θ = 0.013	56.86%	100.00%	100.00%	85.33%

*The threshold value θ is derived when the best classification accuracy is achieved from the RF regressor.*

*^a^RF, Random Forest*.

The obtained results revealed that for the classification of the presence of a particular target tissue, the regression model significantly outperforms the classification model that shares the same learning algorithm. A relatively high threshold value (PIM below 0.064) obtained the best accuracy for the infarction classification when the classification was done with the regressor followed by thresholding.

### 3.3. Impact of Training Set Volume

Supervised machine learning models are sensitive to the volume of training data. To justify if the quantity of cases in the dataset is the bottleneck for the proposal, and to estimate the potential of the predictive models if more training data could be available, a RF regression model of 5,000 estimators was trained several times feeding different quantities of training cases into the model each time. To ensure that the results were comparable, the cross-validation of different folds was applied to control the difference in the training data quantity.

**Figure 4:**
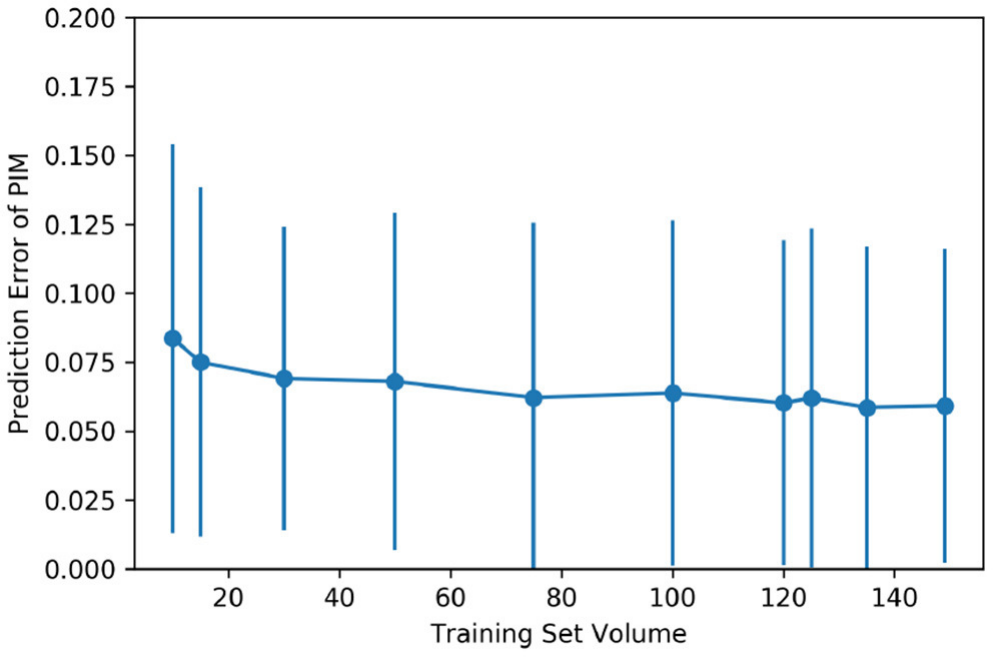
Impact of the training set volume on the mean and the SD of quantification error. The dataset of 150 cases was randomly split into multiple folds to have different amounts of training data. The training set volume ranged from 10 to 149 for each cross-validation.

Figure 4 shows the improvement of the PIM prediction as the training set gets larger. Both the mean error and SD of the PIM quantification decrease along with the increasing evolution of the training set volume. However, as the amount of data increases, the performance improvement becomes less and less obvious.

### 3.4. Performance on Cases of Different Severity of the MI

**Figure 5:**
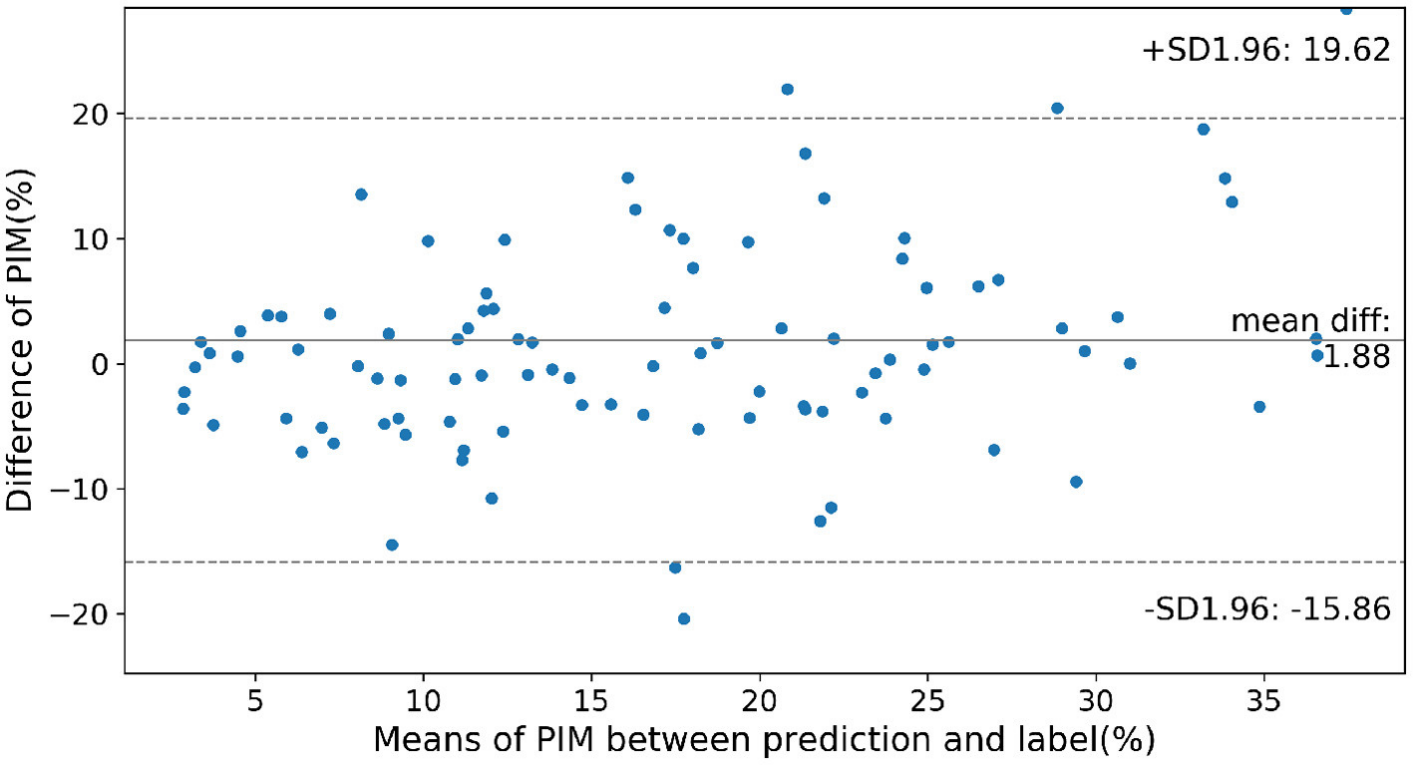
Prediction error on cases of different severity according to the infarction. Normal cases were not considered in this figure. SD, Standard Deviation; mean diff, mean difference.

To show the accuracy of the PIM quantification on the cases of different severity of MI, in Figure 5 the prediction error between the ensemble model and the PIM calculated from the MRI is presented as a Bland-Altman plot only considering the cases with visible MI on DE-MRI. Prediction error rises gradually with the increasing PIM, i.e., the prediction error is larger in the more severe cases.

### 3.5. Importance of Patient Features

The importance of physiological features for the prediction result can be visualized in some regression and classification algorithms. The feature importance for linear or non-linear, regression or classification models, for both the infarction and PMO predictions are presented in Table 5. RF was selected as the non-linear regression and classification models, the ordinary least squares Linear Regressor was the compared linear regression model, and the SVM classifier with linear kernel was the selected linear classifier. The regression models were trained with PIM as the target, while the classification models were trained with the presence of infarction.

**Table 5:** Feature importance to linear and non-linear models for classification and quantification tasks.

**Target**	**Task**	**Model^**a**^**	**Feature importance(%)** ^**b**^																
			**Sex**	**Age**	**SK**	**N-SK**	**F-SK**	**OW**	**HT**	**DB**	**HD**	**ST+**	**Troponin**	**KL1**	**KL2**	**KL3**	**KL4**	**LVEF**	**NTp**
Infarction	Quantification	RFR	2.91	**5.55**	1.04	0.67	0.73	1.06	1.73	0.55	0.61	1.70	**63.01**	0.45	0.26	0.28	0.10	**10.80**	**8.55**
		LR	6.50	3.64	0.00	0.00	0.00	1.17	5.68	1.77	**10.21**	**8.42**	**46.80**	0.00	0.00	0.00	0.00	**11.72**	4.08
	Classification	RFC	**10.30**	**11.90**	2.44	1.65	2.18	2.02	3.21	1.37	2.70	**9.94**	**30.60**	1.18	1.22	0.17	0.10	9.29	9.71
		SVCL	**15.84**	0.93	4.57	5.71	1.14	2.13	1.14	5.22	**21.86**	**14.53**	**21.99**	0.87	0.72	0.50	0.35	0.99	1.50
PMO	Quantification	RFR	1.48	**20.09**	2.58	0.70	1.92	2.31	1.67	0.18	0.17	0.43	**37.38**	1.24	0.46	0.81	0.67	**17.82**	**10.11**
		LR	1.30	**9.40**	0.00	0.00	0.00	2.30	**12.95**	2.02	2.61	10.63	**40.70**	0.00	0.00	0.00	0.00	3.55	**14.54**
	Classification	RFC	4.04	**13.23**	2.57	1.79	2.37	3.42	2.73	1.64	0.88	6.14	**28.88**	1.21	0.99	0.57	0.47	**12.83**	**16.24**
		SVCL	4.90	5.88	1.17	2.27	3.44	0.99	2.13	**9.24**	3.81	**11.71**	**21.75**	5.71	7.55	**8.08**	5.19	5.16	1.02
Mean			5.91	**8.83**	1.80	1.60	1.47	1.93	3.90	2.75	5.36	7.94	**38.38**	1.33	1.40	1.30	0.86	**9.02**	**8.22**

*Categorical features were converted to one-hot encoding. Classification models were trained with the Boolean values, quantification models were trained with PIM. The importance of each model and task was normalized, therefore, the sum of importance was 100%. Higher importance shows a closer relationship between the feature and the disease. Bold indicates the four most important features for each model.*

*^a^RFR, Random Forest Regression; RFC, Random Forest Classifier; LR, Ordinary least squares Linear Regression; SVCL, Support Vector Classifier with Linear Kernel.*

*^b^SK, Smoker; N-SK, Non-smoker; F-SK, Former Smoker; OW, Overweight; HT, Arterial hypertension; DB, Diabetes; HD, History of coronary artery disease; ST+, ST segment elevation according to ECG; KL1, Killip max = 1; KL2, Killip max = 2; KL3, Killip max = 3; KL4, Killip max = 4; LVEF, Left Ventricular Ejection Fraction; NTp, NTProBNP.*

The importance of features in the case of linear models is signed, indicating the direction of influence (positive or negative correlation) of the feature on the prediction. To simplify the results, the absolute normalized importance was kept for the linear models. The mean importance for all the models of each feature was also calculated at the bottom of the table.

The results revealed that for different algorithms and predicted tissues, the feature importance could be slightly inconsistent. Combining all the results, the most important features in order of significance were troponin, sex, history of coronary artery disease and ST+ MI for the infarction, and troponin, age, NTProBNP and ST+ MI for the PMO. It should be noted that the ground truth prediction models learned between the regression and classification models were different, which explained the difference in the feature importance between Random Forest Classifier (RFC) and Random Forest Regressor (RFR).

Furthermore, using RFR as the experimental model, the PIM prediction results were trialed with only selected important features according to Table 5. Results in Table 6 showed that the impact of the feature quantity was inconsistent between the MI and the PMO. The PIM of the MI was generally more reliable with complete features according to its relatively low mean error and lowest SD, while the PMO achieved opposite results. Nevertheless, the high *p*-values (>0.05) between the prediction relying on selected features and the prediction of all 12 features proves that even with the sole feature Troponin, the prediction is already reasonable.

**Table 6:** Percentage of Infarcted Myocardium prediction error with selected important features using Random Forest Regressor.

**Selected features^**a**^**	**PIM**	
	**MI**	**PMO**
Troponin	0.0634 ± 0.0615*	0.0128 ± 0.0226*
Troponin, LVEF	0.0585 ± 0.0620*	0.0122 ± 0.02121*
Troponin, LVEF, NTp, Age	0.0645 ± 0.0598*	0.0145 ± 0.0230*
All 12 features	0.0587 ± 0.0597	0.0149 ± 0.0227

*^a^LVEF, Left Ventricular Ejection; NTp, NTProBNP.*

*If the t-test between the selected features model and the all 12 features model obtains p > 0.05: the difference is not significant.

### 3.6. Comparison With the Types of MI

There is a correlation between the type of MI and our classification results (*r* = 0.603). More specifically, 83.02% of non-infarcted cases were classified as type 2, and 79.38% of infarcted cases where classified as type 1. Moreover, there is a significant difference between the calculated PIM according to the type of MI. Indeed, the mean calculated PIM for type 1 was 0.1775±0.0939 and was 0.0473±0.0410 for type 2 (*p* <10^−5^ obtained from *t*-test).

Since among our examined patient features, ECG and troponin are the most essential ones, according to the definition of MI types, classifications based on sole ECG and troponin were performed. Table 4 revealed the necessity of complementary features: with the same prediction model, the classifications based on 12 pieces of features outperformed the ones based on sole ECG and troponin by 15% for the accuracy of the classification of cases with MI.

### 3.7. Discordant Cases

Even though the results were in general very encouraging, the proposal still provided inaccurate predictions on a few specific cases (Table 7). Incorrect predictions were divided into two categories: the predictions with important difference in the quantification of the PIM and the wrong classifications. Several clinical reasons can explain these mismatches. First, the automatic quantification of the PIM was sometimes underestimated when the troponin was relatively high and associated with ST+ MI but normal ejection fraction (cases 22, 69, and 119). In these cases, discordance between the level of troponin and the LVEF could be observed, certainly due to an overestimation of the ejection fraction acquired at the acute phase. Indeed, a decrease of this value between the acute phase and a measurement carried out at an early moment after the revascularization could be produced, and then a lowest ejection fraction could increase the PIM value. Sometimes the proposal underestimated the PIM despite a relatively high troponin level and low ejection fraction (cases 1, 19, and 105), and it could be considered as a limit of the method. In particular, for the case 105, previous cardiovascular events could explain a high PIM. Sometimes, the results were incomprehensible and maybe the ground truth obtained from DE-MRI underestimated the PIM (as for the cases 7 and 110 were with a high troponin level and altered ejection fraction, a PIM higher than 25% seemed to be correct) or provided a value higher than expected, maybe due to pre-existing necrosis (such as for case 94). Counterexamples could also be found in the classification task, suggesting that the patient could suffer from another cardiovascular disease without uptake on DE-MRI and with preserved cardiac function (such as cases 16, 65, 68, or 117) or without preserved cardiac function (such as cases 34, 114, and 145, suggesting for these cases the presence of chronic disease).

**Table 7:** Cases with incorrect prediction.

**Case^**b**^**	**Feature values** ^**a**^												**GT (%)**	**Prediction (%)**
	**Sex**	**Age**	**TB**	**OW**	**HT**	**DB**	**HD**	**ST+**	**Troponin**	**KL**	**LVEF**	**NTp**		
**Cases with an important PIM Quantification error**														
1	0	32	0	0	0	0	0	1	130	1	35	447	51.64	23.25
7	0	66	0	0	0	0	0	0	200	1	45	532	9.33	25.64
19	0	52	0	1	0	0	0	0	87	3	20	7139	48.04	14.84
22	0	53	0	0	0	0	0	1	170	1	60	43	42.56	23.81
69	1	45	0	0	0	0	0	1	120	1	55	649	39.06	18.62
94	0	61	0	0	1	1	0	1	3.9	1	46	5810	29.75	12.94
105	0	54	2	1	0	0	1	1	25	1	21	4153	46.41	16.52
110	0	49	0	1	0	0	0	1	200	1	45	29	7.54	27.95
119	0	66	2	1	0	0	0	1	73	1	70	159	31.79	9.86
**Wrongly Classified Cases**														
16	0	76	0	1	0	0	0	1	14	1	60	192	0.00	9.32
34	1	78	2	1	0	0	0	1	1.8	1	35	22577	0.00	11.20
65	0	57	1	1	0	0	0	1	19	1	60	71	0.00	8.06
68	0	39	0	1	0	0	0	1	9	1	60	23	0.00	10.40
114	1	54	0	1	1	0	1	0	1	1	45	68	0.00	7.98
117	0	53	1	1	0	0	0	1	83	1	60	94	0.00	18.83
145	1	66	2	1	0	0	0	0	2.5	2	45	6209	14.93	1.38

*Incorrect predictions are presented as the important quantification error and the wrong classification (false positive and false negative). The ground truth (GT) and the prediction values are the PIM. Classification results in the table were obtained by regression model and thresholding.*

*^a^TB, Tobacco; OW, Overweight; HT, Arterial hypertension; DB, Diabetes; HD, History of coronary artery disease; ST+, ST segment elevation according to ECG; KL, Killip max; LVEF, Left Ventricular Ejection; NTp, NTProBNP. For Boolean features, 0 stands for negative (man for Sex) and 1 stands for positive (woman for Sex).*

*^b^Case number of the EMIDEC dataset. Complete data (patient features and MRI) can be accessed on the official website*.

## 4. Discussion

The prediction results are very satisfying for both the quantification task and the classification task. For the quantification task, the ensemble method achieved the best predicted PIM, which showed only 0.056 of error for the PIM comparing with the DE-MRI ground truth. By compared these results with the inter- and intra-observer variation studies done on equivalent data by Chen et al. (35), we can conclude that our method provides results with the same order of error as between experts. Indeed, they found variability of 8.8 and 11% for the intra- and inter-observer variations, respectively. For the classification task, with the RFR followed by thresholding, 133 and 112 of the 150 cases were correctly predicted for the presence of infarction and PMO respectively, representing accuracies of 88.67 and 75.33%. As the data were collected from daily clinical practice and not specifically selected, the prediction accuracy is encouraging given the effect of data collection inaccuracies. Moreover, there is a link between the prediction results and the type of MI defined by the fourth universal definition of MI. Most MI of type 1 were classified as infarcted cases, and the PIM was significantly higher for this type of MI. By using statistical analyses, machine learning brings a more comprehensive interpretation of multiple scalar indicators. It is not straightforward for a standard statistical model to construct such well-fitted non-linear model considering multiple features in a comprehensive manner, and meanwhile to analyze the importance of each feature.

For the automatic PIM quantification, all the regression models involved in the tests obtained satisfying results. Contrary to the findings in the literature studies, boosting models did not significantly outperform the RF, even though the boosting models were computationally intensive. This fact reveals that the predictive model's complexity is not the key issue in improving the prediction performance. The training set volume did have an obvious impact on the prediction error. Indeed, once the volume of data reached approximately 100 cases, the predictive accuracy of the model struggled to increase further as the volume of data increased. Comparing between the regression results with few training samples (*cf*. Figure 4) and the prediction error when the mean ground truth PIM is used to predict all cases (*cf*. Table 3), the smaller prediction error in Figure 4 justifies the efficient use of a very small number of sample data. Meanwhile, the training set volume has a moderate influence on the inference when the training samples are considerable enough. Besides the data volume, the analysis of the discordant cases may reveal the biggest bottleneck in this method for improving performance: ambiguity that originated at the time of data collection. These inaccuracies both increased the bias of the model training and reduced the reliability of the label data during the performance evaluations.

To classify the presence of MI or PMO, the regression models trained with the PIM label followed by thresholding slightly outperformed the classification models trained with the Boolean label (*cf*. Table 4). This fact demonstrated that even for the classification task, the inference results could benefit from richer information provided by the DE-MRI and annotations in the ground truth label. It also justified the advantage of this study, namely a DE-MRI guided physiological, clinical, and paraclinical feature learning system.

When the regression results were taken for the classification task, a relatively high threshold value (PIM of 0.064) for the infarction was observed. This observation reveals that cases close to the critical hyperplane have higher predictive instability. For cases where the regression prediction is around this threshold value, additional complementary clinical exams should be conducted to achieve greater certainty.

In terms of the importance of features for predictive models, the troponin was proved to be the dominant factor to the automatic prediction. This finding echoes the recommendations of The American College of Cardiology/American Heart Association (ACC/AHA) and the European Society of Cardiology (ESC) guidelines that cardiac troponin is the only biomarker for the diagnosis of acute MI due to its superior sensitivity and accuracy (36–39). Age, ST elevation on ECG, LEVF from cardiac TTE and NTProBNP level also demonstrated their obvious contributions. Between the infarction and the PMO, models relied more on the age and NTProBNP for the PMO prediction. This fact indicates a higher relationship between these factors and the presence of the PMO. The history of coronary artery disease only had a noticeable effect on linear models for the infarction prediction. This exception could be explained as the drawback of the linear models: linear models attempt to find a linear combination of the clinical features to distinguish the problem. However, the ideal critical hyperplane for the tasks is apparently not linear, which produces exceptional importance to some features. The observation on the prediction performance with selected features may suggest that the evaluation of the infarction and the available features are well-linked, thus, the PIM for MI increases when more features participate in the prediction. The opposite results on the PMO may indicate the weaker link between it and the available features.

Techniques employing artificial intelligence could become essential to improve cardiologists' work and performances in all aspects of the cardiovascular diseases. In clinical practice, the prediction of the presence or not of a MI and the quantification of myocardial necrosis have a certain interest, first and foremost to confirm or to invalidate a diagnosis and, therefore, to provide information to guide treatment. In the case of an important extent of necrosis with reduction in LVEF, treatment adapted to heart failure can immediately be introduced, a LifeVest wearable defibrillator can be proposed and the doses of diuretics can be better adjusted. Saving time is also important for physicians, and reducing delays with the help of artificial intelligence can allow more patients to benefit from high-performance exams and increase the global quality of care. In addition to the size of the MI, the presence of edema is also important for the prediction of area at risk, potentially quantified by the myocardial salvage index (40). A potential future study could include automatically evaluating the salvage index from physiological, clinical, and paraclinical features, with validation from DE-MRI in combination with T2-weighted images.

Our proposal aims at providing early prediction to better guide patients in the emergency department, which could be the equivalent to risk stratification tools or risk scores. Among them, three well-known risk scores are the GRACE score, the HEART score and the TIMI score (41). These scores are also based on readily available information collected during the admission in the emergency department and their objective is to assess the prognosis as early as possible. The main difference with our approach is that these scores provide a risk stratification and potentially predict benefits from myocardial revascularization performed during initial hospitalization. In our approach, the provided outcomes are less ambitious because we focus on the presence and size of the MI. However PMO, PIM and LVEF are predictors for major adverse cardiac events (MACE) (42), and the first two parameters can be obtained from our method, in addition to LVEF obtained from echography.

One limitation of this study is that the ground truth relies on manual annotation. Chen et al. found an inter-observer variability of 11% on the same type of data (35). This imprecision certainly affects the results of the models and must be reduced. One way to decrease this variability is to consider multi-modal imaging to do the manual contouring and retrieving information from other types of images, such as kinetic images, T1-mapping or T2 mapping at the level of the same slice (acquired at the same moment of the cardiac cycle). Indeed, the myocardial contour could be better defined on cine-MRI, and the boundary of the scar could be clearer on T1-mapping images. However, the major limitation of the proposal is the reliability of the data. Erroneous predictions could be produced because of the measurement inaccuracies of the physiological, clinical, and paraclinical features, or the interference from other diseases like myocarditis, coronary spasms, or the Tako Tsubo syndrome. In future study, to improve the proposal's performance with the given data, the confidence level can be estimated while making the predictions. Since most of the discordant cases can be explained as suspected feature acquisition error, the confidence level can be predicted by analyzing the correlation between features that have strong consistency. Then, in clinical practices, doctors should review the patient reports more thoroughly when the proposed confidence level is relatively low.

## 5. Conclusion

The proposal incorporates basic physiological, clinical, and paraclinical features to provide a rapid and accurate physiological prediction of the severity of acute MI with the help of machine learning approaches. In clinical applications, an automatic assessment of the state of the myocardium and the PIM quantification can be obtained with just these minimal tests including the blood test, ECG and echocardiography. This study can, thus, potentially speed up the disease diagnosis of the acute MI in the emergency cardiology and can also indicate a rethinking of each patient feature's importance for the diagnosis of the disease.

## Data Availability Statement

The original contributions presented in the study are included in the article, further inquiries can be directed to the corresponding author. The anonymized training data can be accessed from http://emidec.com/dataset and the trained models can be download at https://github.com/EMIDEC-Challenge/MI-prediction-from-patient-features.

## Ethics Statement

According to the French law and the Ethical Committee of the University Hospital of Dijon, neither the Ethics Committee approval nor the informed written consent was required.

## Author Contributions

ZC proposed the idea, designed and performed the experiments, and wrote and revised the manuscript. JS designed and performed the experiments and wrote the manuscript. TP provided the cardiology advice and wrote and revised the manuscript. YC prepared the data of the MI types. MS revised the manuscript. TD prepared the experimental data. AL prepared the experimental data, recommended statistical models, revised the manuscript. RC revised the manuscript. All authors contributed to the article and approved the submitted version.

## Funding

This study was supported by Initiatives Science Innovation Territory Economy in Burgundy Franche-Comté project [ISITE-BFC, ANR-15-IDEX-0003]; and the Engineering and Innovation through Physical Sciences, High-technologies, and cross-dIsciplinary research Graduate School [EIPHI, ANR-17-EURE-0002].

## Conflict of Interest

TD works for the Cardiac Simulation and Imaging Software Company (CASIS) that developed the QIR software used in this study. The remaining authors declare that the research was conducted in the absence of any commercial or financial relationships that could be construed as a potential conflict of interest.

## Publisher's Note

All claims expressed in this article are solely those of the authors and do not necessarily represent those of their affiliated organizations, or those of the publisher, the editors and the reviewers. Any product that may be evaluated in this article, or claim that may be made by its manufacturer, is not guaranteed or endorsed by the publisher.

## Footnotes

^1^ https://github.com/EMIDEC-Challenge/MI-prediction-from-patient-features
